# Acute phase proteins as prospective risk markers for arterial stiffness: The Malmö Diet and Cancer cohort

**DOI:** 10.1371/journal.pone.0181718

**Published:** 2017-07-31

**Authors:** Iram Faqir Muhammad, Yan Borné, Gerd Östling, Cecilia Kennbäck, Mikael Gottsäter, Margaretha Persson, Peter M. Nilsson, Gunnar Engström

**Affiliations:** 1 Department of Clinical Sciences, Lund University, Malmö, Sweden; 2 Skåne University Hospital, Malmö, Sweden; University of Colorado Denver School of Medicine, UNITED STATES

## Abstract

**Background and objectives:**

Arterial stiffness plays a significant role in the development and progression of adverse cardiovascular events and all-cause mortality. This observational study aims to explore the relationship between six acute phase proteins namely, ceruloplasmin, alpha-1-antitrypsin, orosomucoid, haptoglobin, complement C3 and C-reactive protein (CRP), and carotid-femoral pulse wave velocity (c-f PWV) in a population-based cohort, and to also explore the effect of low-grade inflammation on the relationship between diabetes and c-f PWV.

**Method:**

The study consisted of participants from the Malmö Diet and Cancer study with data from baseline examinations (1991–1994) and follow-up examinations (2007–2012). Arterial stiffness was measured at follow-up by determining c-f PWV. After excluding participants with missing data, the total study population included 2338 subjects. General linear models were used to assess the relationship between baseline acute phase proteins and c-f PWV.

**Results:**

After adjusting for traditional risk factors the participants in the 4^th^ quartile vs 1^st^ quartile of alpha-1-antitrypsin (geometric mean: 10.32 m/s vs 10.04 m/s) (*p*<0.05), C3 (10.35 m/s vs 10.06 m/s) (*p*<0.05) and CRP (10.37 m/s vs 9.96 m/s) (*p*<0.001) showed significant association with c-f PWV. Diabetes at follow-up was also associated with high c-f PWV, however, this relationship was independent of low grade inflammation.

**Conclusion:**

Alpha-1-antitrypsin, C3 and CRP are associated with arterial stiffness. The results indicate that low grade inflammation is associated with arterial stiffness in addition to established cardiovascular risk factors.

## Introduction

Arterial stiffness is an important risk determinant for adverse cardiovascular events and all-cause mortality [1, 2]. Various studies have shown aortic stiffness to be an independent predictor for all-cause mortality and morbidity in subjects with hypertension, end stage renal disease, type 2 diabetes and the elderly [3–6]. Pulse wave velocity (PWV) has been widely accepted as gold standard for measurement of arterial stiffness [7]. A higher PWV specifies a stiffer aorta, resulting in increased afterload and the subsequent cardiac remodeling and failure [8–10]. Carotid femoral PWV (c-f PWV) measures along the aortic and aorto-iliac pathway and is representative of arterial stiffness [7].

There are multiple risk factors that affect arterial stiffness. Age and blood pressure have been shown to be associated with arterial stiffness [11, 12]. Additionally, many other environmental and genetic factors including dyslipidemia, diabetes [13], elevated heart rate [14], obesity [15] and smoking [16] are often associated with increased arterial stiffness.

There is evidence supporting the fact that inflammation is associated with adverse cardiovascular events, as shown by previous studies [17–19]. It has been demonstrated that acute phase proteins are associated with incidence of myocardial infarction [17], future development of hypertension [18, 19] and incidence of diabetes [20]. Systemic inflammation has also been associated with arterial stiffness [19]. Many studies have explored the association between arterial stiffness and inflammation in subjects with chronic inflammatory conditions such as rheumatoid arthritis [21], systemic lupus erythematosus [22], inflammatory bowel disease [23] and systemic sclerosis [24]. C-reactive protein (CRP) has been shown to be associated with arterial stiffness in an apparently healthy cohort [25] and in subjects with end stage renal disease (ESRD) [26].

A recent cross-sectional study from the Malmö Diet and Cancer study (MDC) reported that waist circumference, raised fasting glucose, insulin resistance and diabetes, and dyslipidemia were associated with increased c-f PWV [27]. Whether the relationship between diabetes and c-f PWV could be modified by low-grade inflammation is unclear. Because of the emerging importance of arterial stiffness in predicting cardiovascular disease risk, a better understanding of the role of inflammatory biomarkers as additional predictors is warranted.

The aim of this observational study is to investigate the association between six acute phase proteins namely, ceruloplasmin, alpha-1-antitrypsin, orosomucoid, haptoglobin, complement C3 and CRP on the one hand and c-f PWV on the other hand in a population-based cohort, followed up for 17 years. Another aim was to study whether the relationship between diabetes and c-f PWV is modified by low-grade inflammation.

## Materials and methods

### Study population

**Fig 1:**
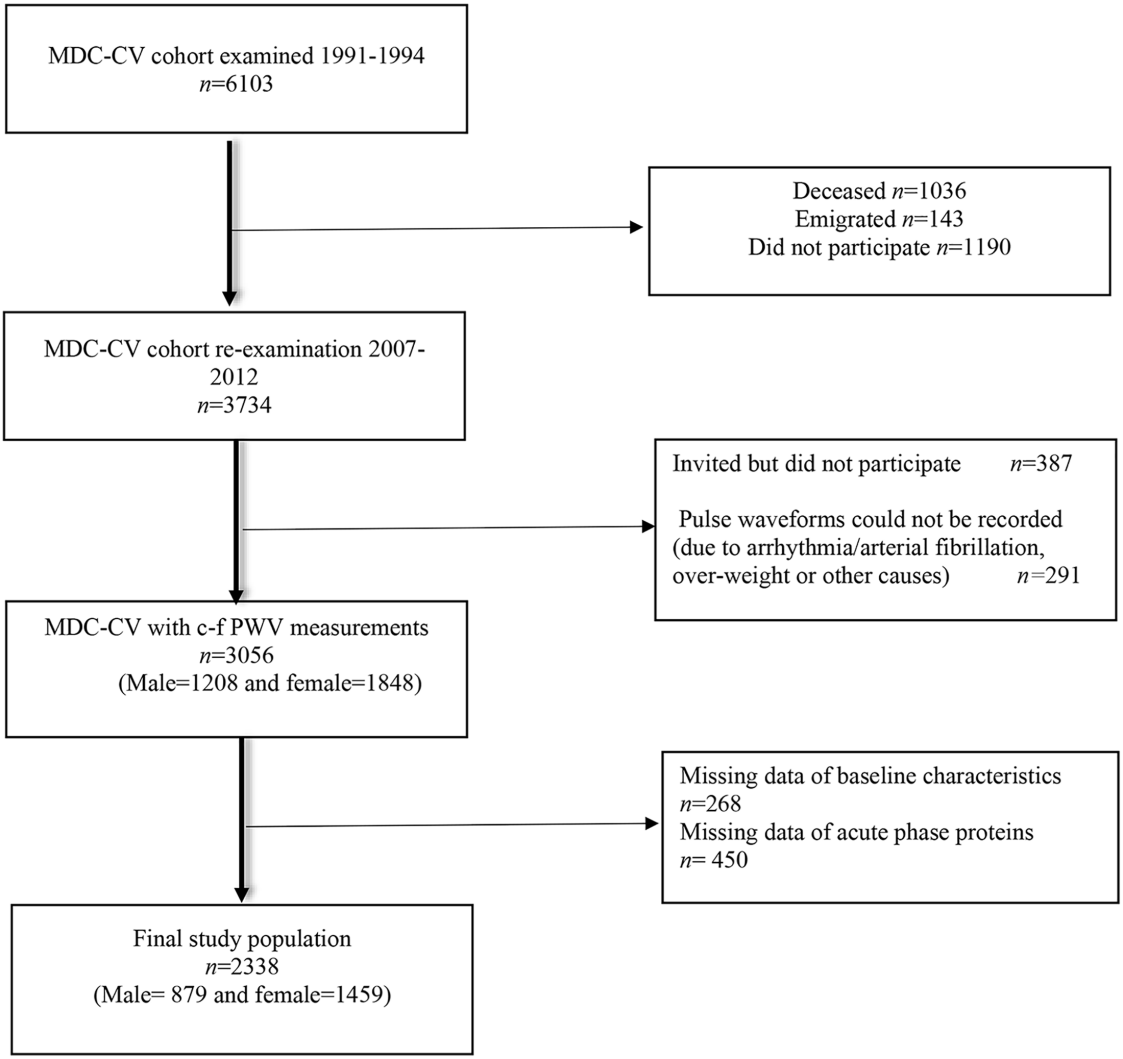
Flow chart of study population (*n = 2338*).

The Malmö Diet and Cancer (MDC) study is a large prospective cohort, compromising of men and women from the city of Malmö, Sweden [28]. A random sample of participants from this cohort was invited between November 1991 and February 1994 to investigate the epidemiology of carotid artery abnormalities, constituting the cardiovascular sub-cohort (n = 6103, 2572 men and 3531 women) [29]. A re-examination of participants from the sub-cohort, who were alive and had not emigrated, was carried out between May 2007 and January 2012. Follow-up data from 3734 participants who attended the re-examination was obtained (76% participation rate). The characteristics of non-attendees of the re-examination have been described elsewhere [30]. Measurements of c-f PWV were available for 3056 participants (Fig 1). Individuals with missing values for baseline characteristics (n = 268), and lacking lab data for one or more acute phase proteins (n = 450) were excluded from the study. The resulting final study population consisted of 2338 participants (879 men and 1459 women). The study population flow chart is illustrated in Fig 1.

The study conforms to the ethical guidelines of the 1975 Declaration of Helsinki. It was approved by the Lund University Ethical Committee (LU 51–90 and LU 532–2006). All the participants provided written informed consent.

### Baseline examinations

Data from baseline examination was collected by means of a self-administered questionnaire, physical examination and collection of blood samples [28]. Information about medical history, smoking habits, use of anti-hypertensive, anti-lipid medication, use of anti-inflammatory medications such as aspirin and steroids was obtained from the questionnaire. Smokers were classified into two categories: non-smokers (i.e., former smokers and never smokers) and current smokers. Blood pressure (mmHg) was measured once, after ten minutes of rest while the subject was in supine position using a mercury-column sphygmomanometer. Waist circumference (in cm) measurements were taken midway between the lowest rib margin and iliac crest. Blood samples were collected after an overnight fast [30]. High-density lipoprotein (HDL) was measured according to standard procedures at laboratory of the University Hospital. Low-density lipoprotein cholesterol (LDL) concentration was calculated according to Friedewald’s formula [31].

Diabetes was defined based on a self-reported physician’s diagnosis of diabetes, use of anti-diabetic medication, or fasting whole blood glucose ≥ 6.1 mmol/L (corresponding to a plasma glucose of 7.0 mmol/L) [32]. Information regarding individuals with rheumatic disease was obtained through a questionnaire. Participants with prevalent cardiovascular disease at baseline were determined according to the questionnaire and hospital registers.

The plasma levels of ceruloplasmin, orosomucoid, haptoglobin, alpha-1-antitrypsin and C3 were analyzed using Cobas c-systems (Roche Diagnostics GmbH, Germany) and have been explained elsewhere [20]. High-sensitive CRP in plasma was analyzed using the Tina-quant^®^ CRP latex assay (Roche Diagnostics, Basel, Switzerland) on an ADVIA^®^1650 Chemistry System (Bayer Healthcare, NY, USA).

### Follow-up examinations

The follow-up examinations were carried out between 2007 and 2012 and comprised of a self-administered questionnaire, blood samples collection and physical examinations. Blood pressure was measured after 5 min rest in supine position using the OMRON M5-1 IntelliSense device. Fasting blood samples were collected for measurement of plasma glucose (p-glucose) using HemoCue (HemoCue AB, Ängelholm, Sweden). Oral glucose tolerance test (OGTT) was conducted following an overnight fast after intake of 75 g of glucose and plasma glucose measurements were done before intake and after 120 min. Fasting plasma glucose of at least 7.0 mmol/L or 2 hour glucose of at least 11.0 mmol/L, verified subsequently by a fasting plasma glucose, was diagnosed as diabetes.

Diabetic cases at follow-up were defined on the basis of six different national and regional diabetes registers. These have been described in detail elsewhere [33]. Additionally, new onset diabetes verified though fasting plasma glucose or OGTT at the rescreening were also included if the date of onset was before the date of measurement of c-f PWV.

### Carotid-femoral pulse wave velocity measurement

The measurements for the c-f PWV were carried out on average 261 days after the first visit in the follow-up examination. They were conducted by using applanation tonometry (SphygmoCor, Atcor Medical, Australia) following a specific study protocol. The measurements were done with the patients in supine position after 5 minutes resting. Pulse curves from the carotid and femoral arteries were obtained with a pressure sensitive probe. For the purpose of calculation, the distance was measured from the suprasternal notch to the umbilicus and the umbilicus to the measuring point at the femoral artery subtracting the suprasternal notch to the measuring point at the carotid artery. ECG was registered simultaneously and the time from the peak of the R-wave on ECG to the foot of the pulse wave at the carotid and femoral arteries was automatically calculated. Each participant had varying number of successful measurements, ranging between one and five. The aim was to obtain three measurements each, which was possible in 86.7% of the cases. Mean c-f PWV was determined from these measurements [27]. Heart rate was described as average heart rate (beats/min) at registration of carotid artery. Blood pressure measurements were also performed just before measuring the c-f PWV using the OMRON M5-1 IntelliSense device. Mean arterial pressure (MAP) was determined by the formula (2 × diastolic pressure + systolic pressure)/3.

Inter-observer variability was determined twice by two technicians in 17 and 13 participants, respectively. Inter-observer difference was 5.0% (± SD4.0) and 7.2% (±SD9.9), respectively.

### Statistical analysis

All statistical analyses were performed using IBM SPSS Statistics version 22 (IBM Corp., Armonk, New York, USA). Variables such as c-f PWV, ceruloplasmin, C3 and CRP were natural log (ln) transformed due to their skewed distribution. Sex-specific quartiles of the acute phase proteins were created, i.e., four quartiles with the same proportion of men and women in each quartile. The baseline and follow-up characteristics of the study population were presented as means ± standard deviation (SD), median (25^th^ -75^th^ percentiles) or percentages. The 2007 European Society of Hypertension-European society of Cardiology (ESH-ESC) guidelines for the management of hypertension proposed a cut off value of c-f PWV of 12 m/s [34]. This value was suggested as a threshold for sub-clinical organ damage. The present study population was categorized into three groups according to their c-f PWV; those with c-f PWV ≥ 12m/s, with c-f PWV <12 and ≥ 8m/s and those with c-f PWV < 8m/s. Comparison between these groups was done for the baseline and follow-up characteristics. To test the differences between these groups, analysis of covariance (ANCOVA) and logistic regression were used for continuous and categorical variables respectively, adjusting for age, sex, heart rate and MAP at follow-up.

Univariate General linear models were used, with c-f PWV as the dependent variable, to assess the association between c-f PWV and quartiles of acute phase proteins. For *Model 1*, the analysis was adjusted for age, sex, heart rate and MAP at follow-up examination. For *Model 2*, further adjustments were done for baseline age, smoking habits, systolic blood pressure, HDL, LDL, waist circumference, presence of diabetes, use of anti-hypertensive medication and use of lipid lowering medication. Interaction terms were introduced to assess any interaction between sex and the acute phase proteins. Univariate general linear model was also used to evaluate the effect of inflammation on the relationship between diabetes at follow-up and c-f PWV by adjusting for the six acute phase proteins and other covariates i.e age, sex, heart rate and MAP at follow-up examination and baseline age, smoking habits, systolic blood pressure, HDL, LDL, waist circumference, use of anti-hypertensive medication and use of lipid lowering medication. For the purpose of these analyses, ln transformed values were used and were transformed back to geometric means to facilitate interpretation of results. A *p*-value of less than 0.05 was regarded as statistically significant.

## Results

### Baseline and follow-up characteristics

The mean follow-up period from the baseline examinations to the c-f PWV measurements was 16.9 ± 1.5 years. The mean age of the study population was 56.0±5.7 and 72.1±5.6 years at the baseline and follow-up examinations, respectively.

**Fig 2:**
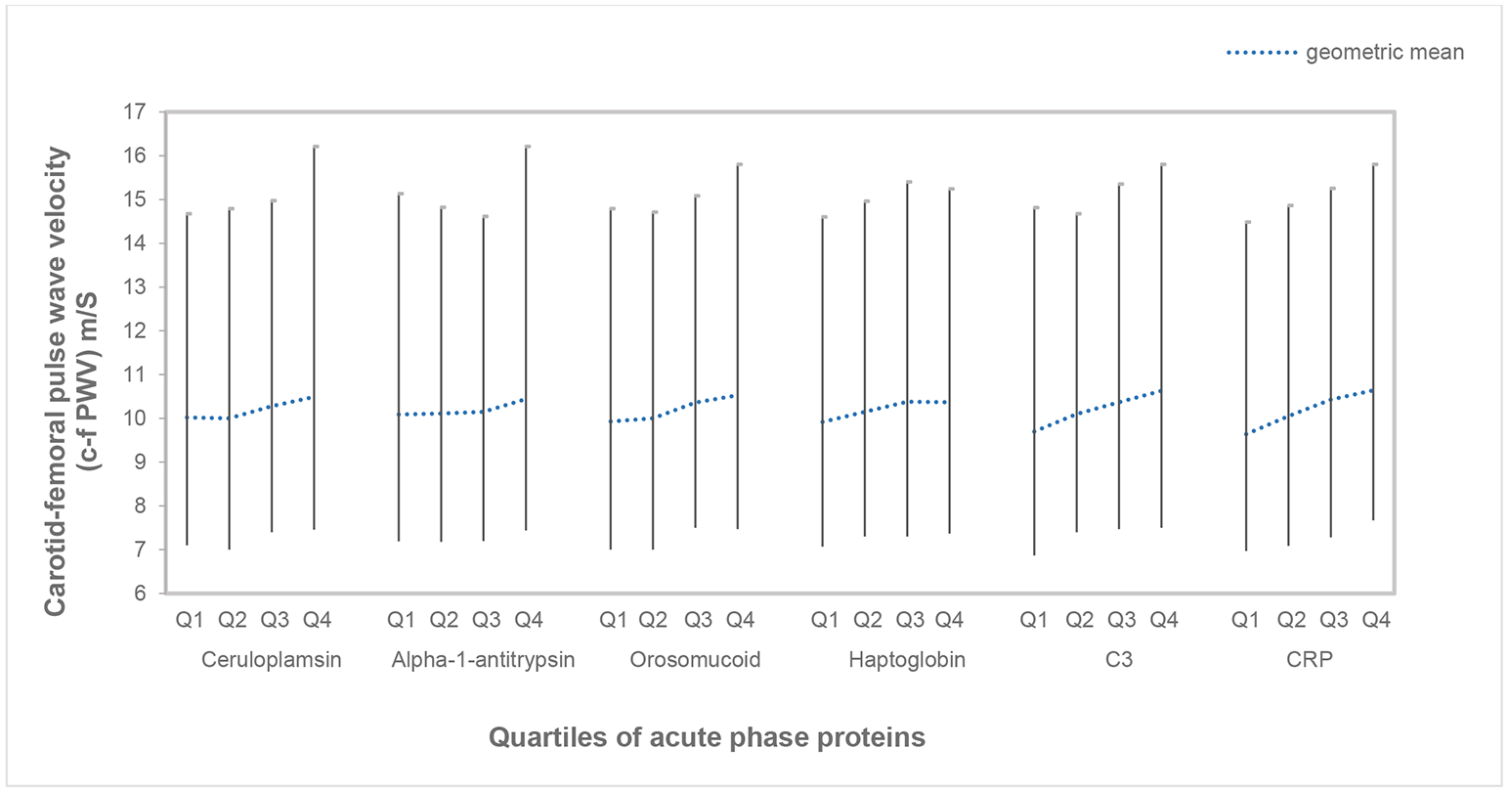
Geometric means and 5th to 95th percentiles of c-f PWV in different quartiles of acute phase proteins.

The characteristics of the study population have been presented in Table 1. The table also displays the comparisons of characteristics between participants with high, intermediate and low arterial stiffness. Those with high arterial stiffness exhibited higher cardiovascular risk, except for smoking, than those with low arterial stiffness. Plasma levels of all the acute phase proteins were higher in those with high arterial stiffness. The geometric means with 5^th^ to 95^th^ percentile of c-f PWV in the different quartiles of the acute phase proteins are presented in Fig 2.

**Table 1:** Follow-up and baseline characteristics of the whole study population (n = 2338) and comparisons of characteristics of participants with high, intermediate and low arterial stiffness.

	*Whole study population (n = 2338)*	*High arterial stiffness (n = 515)*	*Intermediate arterial stiffness (n = 1525)*	*Low arterial stiffness (n = 298)*	p value**
*Follow-up*					
c-f PWV* **(m/s)**	10.03[8.80–11.73]	13.53[12.67–14.83]	9.8[8.97–10.70]	7.4[6.97–7.77]	
**Age (years)**	72.08(±5.56)	75.25(±5.10)	71.68(±5.36)	68.70(±4.53)	
**Heart rate (beats/min)**	62.76(±9.91)	65.27(±10.04)	62.64(±9.96)	59.04(±8.01)	
**Mean arterial pressure (mmHg)**	95.56(±10.49)	100.02(±10.62)	95.44(±9.95)	88.50(±8.89)	
*Baseline*					
**Age (years)**	55.97(±5.65)	59.21(±5.27)	55.56(±5.44)	52.50(±4.53)	0.112
**Smoking {current smokers [n (%)]}**	398(17)	63(12.2)	273(17.9)	62(20.8)	0.230
**Systolic blood pressure (mmHg)**	137.58(±17.64)	145.48(±17.67)	136.33(±16.89)	130.28(±16.55)	<0.001
**Waist (cm)**	81.25(±11.60)	84.99(±12.29)	80.55(±11.31)	78.41(±10.32)	<0.001
**HDL cholesterol(mmol/L)**	1.43(±0.37)	1.38(±0.38)	1.44(±0.36)	1.46(±0.35)	0.002
**LDL cholesterol (mmol/L)**	4.09(±0.96)	4.30(±0.99)	4.05(±0.94)	3.91(±0.95)	0.015
**Diabetes [n (%)]**	121(5.2)	60(11.7)	55(3.6)	6(2)	<0.001
**Lipid lowering drug medication [n (%)]**	43(1.8)	17(3.3)	20(1.3)	6(2)	0.234
**Anti- hypertensive medication [n (%)]**	272(11.6)	95(18.4)	158(10.4)	19(6.4)	<0.001
Ceruloplasmin* **(g/L)**	0.49[0.43–0.56]	0.50[0.43–0.57]	0.48[0.43–0.56]	0.48[0.42–0.55]	0.009
**Alpha-1-antitrypsin(g/L)**	1.18(±0.25)	1.18(0.25)	1.18(0.24)	1.16(0.27)	0.321
**Orosomucoid(g/L)**	0.68(±0.20)	0.71(0.20)	0.67(0.19)	0.64(0.19)	<0.001
**Haptoglobin(g/L)**	1.25(±0.51)	1.31(0.51)	1.23(0.50)	1.20(0.52)	0.025
Complement C3*(g/L)	1.45[1.28–1.64]	1.54[1.35–1.72]	1.43[1.28–1.62]	1.37[1.19–1.58]	<0.001
CRP* **(mg/L)**	1.10 [0.60–2.30]	1.50[0.70–2.80]	1.10[0.60–2.20]	0.80[0.50–1.60]	<0.001

Values are expressed as means (±SD) or percentages unless specified elsewise. High arterial stiffness: c-f PWV ≥12 m/s; intermediate arterial stiffness: ≥8 m/s and <12m/s; Low arterial stiffness: c-f PWV <8 m/s

*median [25^th^–75^th^ percentile].

** p—values for differences between groups with high, intermediate and low arterial stiffness after adjustment for age, sex heart rate and mean arterial pressure (MAP).

### Relationship between acute phase proteins and c-f PWV

When the different quartiles of the acute phase proteins were compared, the association between all the acute phase proteins and c-f PWV were significant for the participants in the 4^th^ quartile vs the 1^st^ quartile in *Model 1* as shown in Table 2. After further adjustments in *Model 2*, the c-f PWV remained significantly higher for participants in the 4^th^ quartile vs 1^st^ quartile of alpha-1-antitrypsin (geometric mean: 10.32 m/s vs 10.04 m/s) (*p*<0.05), C3 (10.35 m/s vs 10.06 m/s) (*p*<0.05) and CRP (10.37 m/s vs 9.96 m/s) (*p*<0.001). The *p*-values for trend across quartiles of acute phase proteins are shown in Table 2. The interaction terms between sex and the acute phase proteins were not significant.

**Table 2:** Relationships between the acute-phase proteins and c-f PWV.

***Model 1***					
	***Geometric means (n)***	***Geometric means (n)***	***Geometric means (n)***	***Geometric means (n)***	
	**Q1**	**Q2**	**Q3**	**Q4**	***p* for trend**
**Ceruloplasmin**	10.13 (558)	10.04 (606)	10.22 (597)	10.40* (577)	0.004
**Alpha-1-antitrypsin**	10.05 (595)	10.12 (505)	10.26 (673)	10.34** (565)	0.004
**Orosomucoid**	10.05 (645)	10.00 (531)	10.21 (577)	10.52*** (585)	<0.001
**Haptoglobin**	10.04 (633)	10.20 (562)	10.21 (559)	10.35** (584)	0.006
**Complement-C3**	9.89 (577)	10.08 (595)	10.27*** (586)	10.55*** (580)	<0.001
**CRP**	9.84 (534)	10.04 (610)	10.36*** (607)	10.52*** (587)	<0.001
***Model 2***					
	**Q1**	**Q2**	**Q3**	**Q4**	***p* for trend**
**Ceruloplasmin**	10.18	10.05	10.21	10.36	0.035
**Alpha-1-antitrypsin**	10.04	10.16	10.27*	10.32*	0.007
**Orosomucoid**	10.20	10.03	10.18	10.37	0.058
**Haptoglobin**	10.12	10.26	10.17	10.25	0.440
**Complement-C3**	10.06	10.14	10.24	10.35*	0.012
**CRP**	9.96	10.10	10.33**	10.37***	<0.001

* *p*<0.05

** *p*<0.01

***p<0.001

Ln transformed values were used to calculate the p-values with Q1 as the reference group. Model 1: Adjusted for age, sex, heart rate, mean arterial pressure (MAP) at follow-up. Model 2: Adjusted for age, sex, heart rate, mean arterial pressure (MAP) at follow-up, and baseline age, smoking habits, systolic blood pressure, HDL, LDL, waist circumference, diabetes, use of anti-hypertensive medication and use of lipid lowering medication

C-f PWV was also compared in individuals with and without diabetes at the follow-up examination. At the re-examination, 305 individuals had diabetes and 2033 were non-diabetic. As expected, diabetes was significantly associated with c-f PWV (geometric means: 11.16 m/s vs 10.06 m/s) (*p*<0.001). After adjusting for the acute phase proteins and other covariates the c-f PWV remained significantly higher for individuals with diabetes vs those without diabetes (geometric means: 10.61 m/s vs 10.14 m/s) (*p*<0.001).

As arterial stiffening increases in individuals with rheumatic and cardiovascular disease, a sensitivity analysis was performed after excluding those with prevalent rheumatic and cardiovascular disease at re-examination. A total of 48 individuals had rheumatic disease (according to questionnaire) and 27 had prevalent cardiovascular disease (according to questionnaire and hospital registers) at baseline. At re-examination, the number of participants with rheumatic and cardiovascular disease was 88 and 171 respectively.

Regarding anti-inflammatory medication, 0.3% (n = 7) and 3.5% (n = 82) of the subjects were taking steroids and aspirin respectively at baseline. These were also excluded for the purpose of the sensitivity analysis. The results showed that the association was still significant between alpha-1-antitrypsin, C3, CRP and arterial stiffness.

## Discussion

The results from this large, observational, population-based study show that acute phase proteins alpha-1-antitrypsin, C3 and CRP are associated with future c-f PWV, even after controlling for traditional cardiovascular risk factors. This suggests that low grade inflammation may play a role in the pathogenesis of arterial stiffening, hence contributing to increased cardiovascular risk. Since inflammation is associated with increased incidence of diabetes, we also hypothesized that inflammation could modify the relationship between diabetes and arterial stiffness. We could confirm that diabetes at follow-up was strongly associated with c-f PWV, however, this relationship was independent of baseline low-grade inflammation in this study.

The mechanism as to how these inflammatory biomarkers modify arterial stiffness is unclear. Numerous studies have looked at the link between inflammation and arterial stiffness, but these have consisted mostly of study populations with chronic inflammatory diseases [21–24]. It should be noted that inflammatory markers are associated with other traditional risk factors of arterial stiffness such as obesity and diabetes [13, 15]. However, as adjustment for these risk markers was done in the analyses, it can be postulated that inflammation effects arterial stiffness through a mechanism besides the one linked to these established risk markers. It is interesting to note that one study comprising of another chronic condition associated with systemic inflammation, namely chronic obstructive pulmonary disease (COPD), demonstrated that arterial stiffness in COPD was not related to systemic inflammation [35]. This is in contrast to the above mentioned studies. Although this is interesting, low grade inflammation could still have a role in arterial stiffness. One explanation could be that levels of inflammatory markers are more variable in patients with COPD than in healthy volunteers.

Alpha-1-antitrypsin is a proteinase inhibitor with the primary function of inhibiting proteolytic enzymes like neutrophil elastase. Hence, it plays a role in maintaining the vascular structure. Our results show that there is association between alpha-1-antitrypsin and arterial stiffness after adjustment for the traditional risk factors. This is an interesting finding as previous studies have assessed the relationship between alpha-1-antitrypsin and various cardiovascular diseases with conflicting results [36, 37]. Most of these findings are, however, related to associations with decreased levels of alpha-1-antitrypsin. Ducker *et al*. demonstrated that a group of patients with alpha-1–antitrypsin deficiency and related chronic obstructive pulmonary disease had increased aortic stiffness [36]. Looking at the different alleles for the of the alpha-1 antitrypsin (*SERPINA1*) gene, a study undertaken by Dahl *et al*. concluded that alpha-1-antitrypsin deficiency in *ZZ* and *MZ* genotypes is associated with reduced blood pressure in individuals with ischemic heart disease (IHD) as compared to *MM/MS* genotypes. Additionally, *MZ* heterozygosity was associated with reduced risk of ischemic cerebrovascular and IHD [37]. One plausible explanation could be that raised alpha-1-antitrypsin levels reflect a compensatory mechanism which reduces the adverse effects of an ongoing proteolytic activity and degradation of elastin. As loss of elastin is central to the stiffening process [38], the proteolytic activity could cause decreased elasticity of the vessel wall and hence, leading to increased arterial stiffness.

It has also been observed in the present study that C3 is associated with c-f PWV. To our knowledge only one previous study has looked at the association between C3 and c-f PWV. The study included middle aged women with Systemic Lupus Erythematosus (SLE) and demonstrated positive association between C3 and CRP on the one hand with aortic stiffness on the other [39]. This is in accordance with our results. C3 has been known to play a role in cardiovascular disease development and hypertension [40, 41]. However, the association between C3 and arterial stiffness has not been widely explored. Shields *at al*. proposed a mechanism to explain the association between C3 and arterial stiffness [42]. Their study suggested that complement factors C3 and C4, in the absence of plaque formation, target the adventitia and bind to the elastin and collagen fibers in the vascular wall, hence contributing to the progression of arterial stiffness.

The results from the present study show that CRP levels and c-f PWV are associated. These findings are supported by studies that have shown association of CRP with arterial stiffness in apparently healthy individuals [25, 43]. Still further yet, in the Rotterdam study, Mattace *et al*. demonstrated that there is an association between CRP and arterial stiffness in older adults [44]. Results from cross-sectional studies have been varying, but two large prospective studies have shown the association of inflammation and aortic stiffness. The Whitehall II study and the Caerphilly prospective study showed that there is association between CRP and aortic stiffness [45, 46]. In contrast, a study by Amar *et al*. showed that although CRP and peripheral pulse pressure were associated, this was not seen with aortic stiffness [47]. It has been proposed that CRP can impact on vascular stiffness by means of its role in endothelial dysfunction and thereby lead to disruption of structural regulation of arteries and consequently arterial stiffness [48]. However, Schumacher *et al*. explored the association between circulating CRP levels, the CRP genotype and PWV. Interestingly, the study concluded that although there exists an association between circulating levels of CRP and c-f PWV, there is no causal relationship between CRP and arterial stiffness [49].

There is substantial evidence suggesting association between diabetes and arterial stiffness [13, 50, 51]. Arterial stiffness has also been shown to increase during the pre-diabetes stage [52]. Inflammation is commonly linked to both diabetes and arterial stiffness. It has recently been demonstrated in the present cohort that high levels of acute phase proteins are associated with the development of diabetes [20]. The results of the present study showed that those with diabetes had higher c-f PWV regardless the presence of inflammation. This implies that the effect of diabetes, and factors linked to the diabetes state, is substantial in the progression of arterial stiffness.

One of the strengths of this study is that it is a large population-based cohort with a long follow-up period. Although it can be argued that the final study population was health-selected as many individuals with poor health died before the re-examination, it has been demonstrated that the study cohort was representative of the population in terms of baseline characteristics, when examined through a mailed health survey (participation rate 75%) [28]. Another strength of the study is that the analysis was adjusted for majority of major risk factors at baseline and follow-up. Hence, the results indicate that systemic inflammation is a potential determinant for arterial stiffness. However, one of the limitations of the study is that the c-f PWV and plasma levels for acute phase proteins were measured only once and separated in time. No further measurements were carried out at follow-up examination to evaluate the change in plasma levels over this period. It has been shown that levels of inflammatory markers are quite stable over time in healthy individuals [53, 54]. It has been reported that the variability of CRP over a one-year period is comparable to that of cholesterol [53]. Nevertheless, it can be assumed that a random misclassification would reduce the associations between c-f PWV and inflammation.

Even though c-f PWV is a valid measure of the arterial stiffness in the aortic and aorto-iliac vascular tree, it should be acknowledged that c-f PWV does not reflect the heterogeneity which occurs in various primary and secondary branches. A recent study by Weir-McCall et al. demonstrated differences in carotid-femoral measured PWV and MRI measured aortic PWV, which can be for the most part be explained by use of simple surface measurements [55]. Nevertheless, distance estimation has been performed in a standardized way in this study. It should also be acknowledged that the statistical power was limited when comparing patients with diabetes, and it is possible that a larger sample would result in significant differences. It should also be noted that these patients with diabetes were mostly treated by a number of drugs (also anti-diabetic drugs) and this could have reduced the prediction of acute phase reactants.

In conclusion, this prospective population-based cohort study shows that alpha-1-antitrypsin, C3 and CRP are associated with c-f PWV and, therefore, arterial stiffness. This observation suggests that inflammation may be involved in the pathogenesis of arterial stiffening. The results also indicated that individuals with diabetes had increased c-f PWV. However, this relationship was not explained by raised inflammatory proteins. A better understanding of the underlying mechanism of association between low grade inflammation and arterial stiffness is necessary, due to its important implications on future diagnosis, risk stratification, and potential therapeutic approach of cardiovascular diseases.
